# Primary nasopharyngeal tuberculosis: a case report

**DOI:** 10.1186/s12879-016-1449-7

**Published:** 2016-03-11

**Authors:** Yoshio Nakao, Rei Shibata, Toyoaki Murohara, Tohru Tanigawa

**Affiliations:** Department of Otolaryngology, Yoshida General Hospital, Hiroshima, Japan; Department of Advanced Cardiovascular Therapeutics, Nagoya University Graduate School of Medicine, Nagoya, 466-8550 Japan; Department of Cardiology, Nagoya University Graduate School of Medicine, Nagoya, Japan; Department of Otorhinolaryngology, Aichi Medical University, Nagakute, Aichi 480-1195 Japan

**Keywords:** Nasopharyngeal, Nasolaryngoscopy, Postnasal drips, Tuberculosis

## Abstract

**Background:**

The occurrence of nasopharyngeal tuberculosis is rare even in areas where tuberculosis is endemic. Here, we report a case of rare primary nasopharyngeal tuberculosis, promptly evaluated by nasolaryngoscopy.

**Case presentation:**

A 78-year-old woman presented with postnasal drip and a cough of 1-month duration. Endoscopic examination of the nasopharynx revealed irregular mucosal thickening of the right lateral and posterior wall of the naso (epi)-pharynx, which was covered with yellow discharge presenting as postnasal drip. Computed tomography (CT) demonstrated enhanced soft tissue area in the right lateral and posterior wall of the nasopharynx. Bacteriological examination from a nasopharyngeal swab revealed that staining for acid-fast bacilli was positive and the quenching probe PCR test was positive for *Mycobacterium tuberculosis*. Histopathological examination from the thickening nasopharyngeal mucosa revealed granulomatous formation with caseous necrosis. Ziehl-Nielsen staining directly could detect acid-fast bacilli. Chest X-ray and CT scan ruled out the pulmonary tuberculosis. Base on these findings, we diagnosed it as primary nasopharyngeal tuberculosis. After six months anti-tuberculous therapy, the patient’s symptoms had completely disappeared. Nasolaryngoscopic examination and CT image after 6 months post therapy revealed a normal nasopharynx with complete resolution of the lesion.

**Conclusion:**

We recommend endoscopic examination for patients suffering from chronic postnasal drips to avoid inappropriate diagnosis.

## Background

Mild symptoms related to the nasopharynx can be easily overlooked, and may present themselves as serious medical conditions at a later stage. When symptoms become chronic, nasolaryngoscopy is used to rule out serious medical conditions such as malignancies. Nasolaryngoscopy is easy to learn, safe, painless, convenient to perform, and readily accepted by patients. Furthermore, it also allows direct visualization and evaluation of both the lesion and the nasopharynx and larynx anatomy, under local anesthesia. It thus allows for early diagnosis and treatment in a primary care center by reducing unnecessary referral [1, 2].

The occurrence of nasopharyngeal tuberculosis is rare even in areas where tuberculosis is endemic. Our literature search for primary nasopharyngeal tuberculosis revealed that very few cases have been published thus far [3–5]. Here, we report a case of rare primary nasopharyngeal tuberculosis, promptly evaluated by nasolaryngoscopy.

## Case presentation

**Fig. 1:**
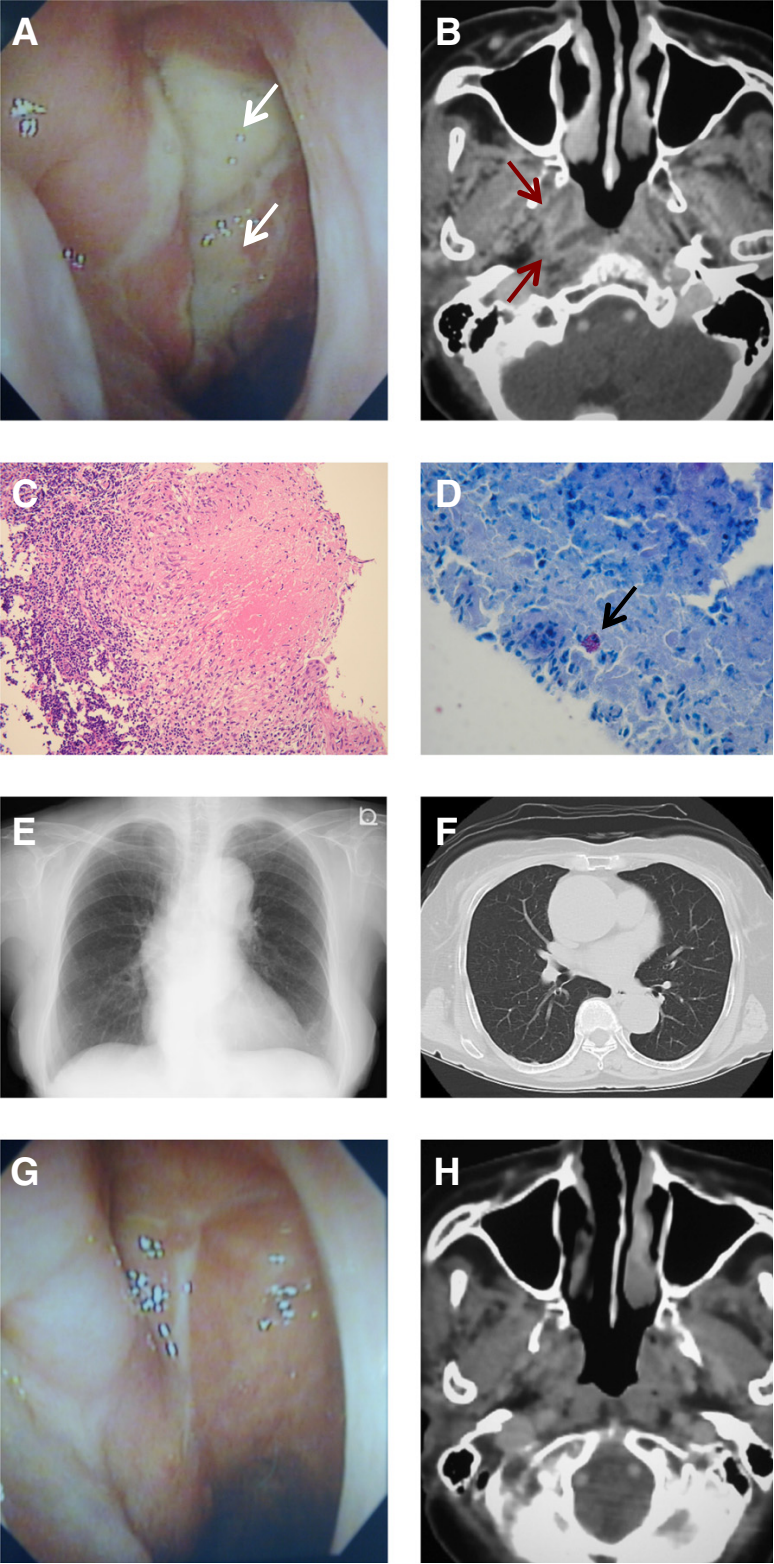
Nasopharyngeal tuberculosis. **a** Nasolaryngoscopic examination showing irregular mucosal thickening of the right lateral and posterior wall of naso (epi)-pharynx, which was covered with yellow discharge presenting as postnasal drip (arrow). **b** Computed tomography (CT) of the nasopharynx. CT images showing the enhanced soft tissue area in the right lateral and posterior wall of the nasopharynx (arrow). **c** Histopathological examination of the thickened nasopharyngeal mucosa showing granuloma formation with caseous necrosis. **d** Ziehl-Nielsen staining from biopsy specimens with acid-fast bacilli (arrow). Chest X-ray **e** and CT scan **f** ruled out the pulmonary tuberculosis. **g** and **h** Nasolaryngoscopic examination and CT image after 6 months anti-tuberculous therapy showing a normal nasopharynx with complete resolution of the lesion

A 78-year-old woman presented with postnasal drip and a cough of one month duration. There were no cervical lymph nodes palpable. General symptoms of tuberculosis (evening rise fever and weight loss) were not observed. Endoscopic examination of the nasopharynx revealed irregular mucosal thickening of the right lateral and posterior wall of the naso (epi)-pharynx, which was covered with yellow discharge presenting as postnasal drip (Fig. 1a). Computed tomography (CT) demonstrated enhanced soft tissue area in the right lateral and posterior wall of the nasopharynx (Fig. 1b). Bacteriological examination from a nasopharyngeal swab revealed that staining for acid-fast bacilli was positive (Gaffky-1) and the quenching probe PCR test was positive for *Mycobacterium tuberculosis*. Moreover, QuantiFERON-TB Gold was positive. Histopathological examination from the thickening nasopharyngeal mucosa revealed granulomatous formation with caseous necrosis (Fig. 1c). Ziehl-Nielsen staining directly could detect acid-fast bacilli (Fig. 1d). Indigenous bacterial flora was only detected in sputum culture. Chest X-ray and CT scan ruled out the pulmonary tuberculosis (Fig. 1e and f). Base on these findings, we diagnosed it as primary nasopharyngeal tuberculosis. After six months anti-tuberculous therapy, the patient’s symptoms (sore throat and postnasal drip) had completely disappeared. Repeat bacteriological examination of the nasopharyngeal swab at the same time was negative for acid-fast bacilli. The quenching probe PCR test was also negative for *Mycobacterium tuberculosis*. Nasolaryngoscopic examination and CT image after 6 months post therapy revealed a normal nasopharynx with complete resolution of the lesion (Fig. 1g and h).

## Conclusions

Nasopharyngeal tuberculosis is commonly associated with cervical lymphadenopathy [3, 4]. In the present case, we did not find any palpable or enlarged lymph nodes on clinical and CT scan examination, respectively. Although nasopharyngeal tuberculosis may present itself in many ways, signs and symptoms can be missed easily if they are less obvious [5]. Such symptoms in the form of chronic cough and postnasal drip were found in the present case, which is rather unusual. We wish to highlight the fact that considering the age of the patient and chronicity of symptoms, this case could have been diagnosed much earlier if a nasolaryngoscopy had been performed. As early as 1988, a study of nasolaryngoscopy has shown that family physicians can perform the procedure with a mean examination time of 4.6 min with good patient tolerability [6]. Although, primary nasopharyngeal tuberculosis is extremely rare [4], we recommend endoscopic examination by family physicians at primary care centers for patients with chronic nasopharyngeal complaints.

### Consent

Written informed consent was obtained from the patient for publication of this Case report and any accompanying images. A copy of the written consent is available for review by the Editor of this journal.

## Abbreviations

CT: computed tomography
PCR: polymerase chain reaction
